# Cellular Models of Aging and Senescence

**DOI:** 10.3390/cells14161278

**Published:** 2025-08-18

**Authors:** Byunggik Kim, Dong I. Lee, Nathan Basisty, Dao-Fu Dai

**Affiliations:** 1 Division of Cardiology, Department of Medicine, School of Medicine, Johns Hopkins University, Baltimore, MD 21205, USA; bkim96@jhmi.edu; 2 Translational Gerontology Branch, National Institute on Aging, NIH, Baltimore, MD 21224, USA; nathan.basisty@nih.gov; 3 Department of Pathology, School of Medicine, Johns Hopkins University, Baltimore, MD 21205, USA

**Keywords:** cellular aging, senescence, induced pluripotent stem cells (iPSCs), epigenetic reprogramming, mitochondrial dysfunction, senolytics, progeroid syndromes, in vitro models, cardiovascular aging, neurodegeneration

## Abstract

Aging, a state of progressive decline in physiological function, is an important risk factor for chronic diseases, ranging from cancer and musculoskeletal frailty to cardiovascular and neurodegenerative diseases. Understanding its cellular basis is critical for developing interventions to extend human health span. This review highlights the crucial role of in vitro models, discussing foundational discoveries like the Hayflick limit and the senescence-associated secretory phenotype (SASP), the utility of immortalized cell lines, and transformative human induced pluripotent stem cells (iPSCs) for aging and disease modeling and rejuvenation studies. We also examine methods to induce senescence and discuss the distinction between chronological time and biological clock, with examples of applying cells from progeroid syndromes and mitochondrial diseases to recapitulate some signaling mechanisms in aging. Although no in vitro model can perfectly recapitulate organismal aging, well-chosen models are invaluable for addressing specific mechanistic questions. We focus on experimental strategies to manipulate cellular aging: from “steering” cells toward resilience to “reversing” age-related phenotypes via senolytics, partial epigenetic reprogramming, and targeted modulation of proteostasis and mitochondrial health. This review ultimately underscores the value of in vitro systems for discovery and therapeutic testing while acknowledging the challenge of translating insights from cell studies into effective, organism-wide strategies to promote healthy aging.

## 1. Introduction

The quest to understand and influence the aging process is arguably one of humanity’s oldest biological inquiries, dating back to as early as 2700 BC [1]. Aging is not a singular event. Instead, it is a complex multifaceted process involving many molecular and cellular changes that accrue over time in an organism. Understanding the fundamental processes that underpin aging is considered a prerequisite for enhancing human health span and mitigating the global burden of age-related pathologies.

Longevity is ultimately determined by an accumulation of damage and dysfunction at the molecular and subcellular levels [2,3,4]. These so-called hallmarks of aging include diverse, interacting processes such as increasing genetic instability, pervasive epigenetic alterations, accumulation of cellular and genomic damage, a progressive loss of proteostasis that leads to misfolded proteins, altered autophagy, declining mitochondrial and metabolic function, and general organelle dysfunction [3,5,6].

The scientific community relies heavily on experimental models to study and intervene in the aging process. Aging is too complex to be represented by a single approach due to its heterogeneity, which includes a wide spectrum of changes at the molecular and organismal levels. Many experimental approaches in aging research generally fall under a “steering” category. In these research models, cells or organisms are like vehicles on the established and unavoidable road of natural aging path. Scientists either hit the gas to accelerate the aging process to gain a deeper understanding or more often apply the brake or even shift into reverse with interventions to decelerate or even partially rejuvenate the changes of aging. For instance, treatments that selectively clear senescent cells effectively steer the system toward a more youthful state. Other interventions that alter mTOR, proteostasis, and autophagy pathways are examples of steering. Rather than offering precise control over cells’ age-related dysfunction, these approaches tend to broadly shift the cells’ state to a more youthful status. This approach is central to current translational aging research.

Looking to future research, a more ambitious approach for aging intervention is selectively defining biological age. For example, transiently expressing a specific gene set can rewind cells’ epigenetic clock and restore youthful function. Rather than completely resetting the cells’ identity, it allows a cell to become functionally younger while maintaining its cellular identity. This represents the initial concrete stride towards specifically defining a more youthful age without regressing to a fetal or developmental stage. However, it remains an ambitious future goal to be able to achieve the precise selected youthful and functional state as an intervention against the aging process.

**Figure 1:**
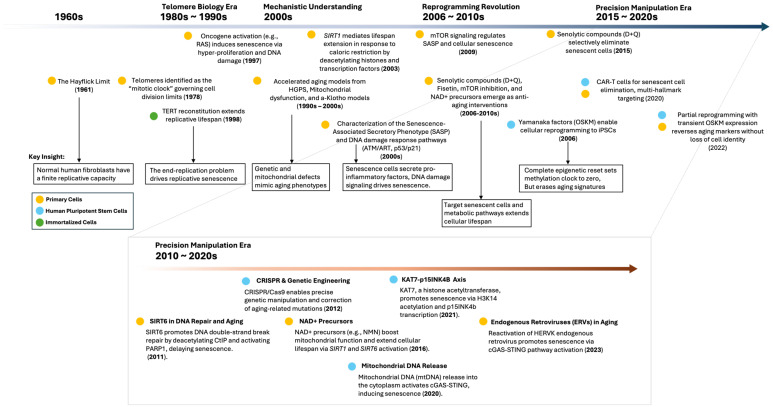
Timeline of major findings from in vitro cellular models in aging. This figure illustrates the evolution of cellular aging research from the 1960s to 2025, highlighting key discoveries and interventions derived from in vitro cellular models. The timeline is divided into five eras: foundation era (1960s), telomere biology era and mechanistic understanding (1980s–2000s), reprogramming revolution (2006–2010s), precision manipulation era (2010–2020s), and precision manipulation era (2015–2020s to 2025).

Many exciting cell culture findings in biomedical research have not led to any effective therapies, a fact that often leads to disappointment. However, this does not diminish the importance of these cellular models. Instead, it defines their true purpose: they are valuable for creating foundational knowledge, which needs to be validated in other model systems to develop definitive therapeutic solutions. Related to this, although no in vitro model can perfectly capture the complexities of organismal aging, these models still have great merit. Cell- or organoid-based systems, as well as in vitro tissue engineering approaches, are critical to deconstructing signaling pathways and molecular events that contribute to the aging phenotype. They provide the controlled environment necessary to untangle the web of cause and effect, thread by thread. In line with this emphasis on the strength of controlled experimental systems, this review primarily focuses on in vitro models in the context of physiological aging and age-related pathologies, in particular cardiovascular and neurodegenerative diseases. We discuss how in vitro systems are used to model aging and for the discovery and testing of therapies to extend health span. Some of the crucial findings from cellular models are outlined in Figure 1.

## 2. Cellular Models of Aging—Artificial Stressors

A major aim in aging research is to separate the unavoidable progression of chronological time from the harmful decline in function with biological age. One advantage of cell models is that by taking cells out of their natural, complex environment, we eliminate many sophisticated homeostatic, protective, and repair mechanisms within organ systems. In this sense, cultured cells may represent more “extreme” or “unbuffered” conditions, where nutrient availability is standardized, waste removal is periodic, complex intercellular communication is typically reduced (unless specifically engineered), and systemic influences like hormones or nuanced immune surveillance are absent [7,8]. Consequently, intrinsic cellular aging processes or responses to age-related stressors can be easily manipulated to resemble the natural progression of aging.

The history of in vitro aging research began with studies by Hayflick and Moorhead in the 1960s using primary human diploid fibroblasts. This work demonstrated that normal somatic cells possess a finite replicative capacity before entering replicative senescence, a phenomenon called the “Hayflick limit” [9]. The discovery was foundational, directly linking mitotic activity to cellular replicative lifespan. Subsequent research using primary fibroblasts has been pivotal in understanding that telomere attrition—the progressive shortening of chromosome ends with each cell division due to the end-replication problem—is a key mechanism that triggers this replicative senescence [10,11,12]. By understanding the process of replicative lifespan, it is necessary to realize that chronological time does not fully capture the state of aging in cells. Recent studies reveal the concept of the biological clock, i.e., physiological state and functional capacity of the cells, which can be measured by epigenetic markers. This biological or epigenetic clock may represent the progress of aging process even better.

**Figure 2:**
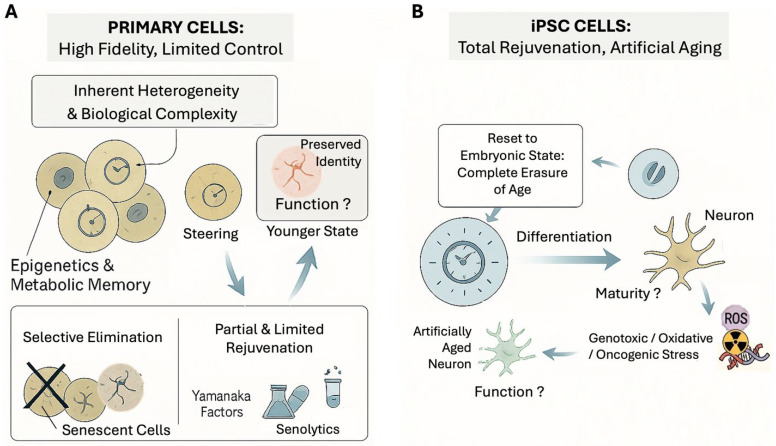
Two cellular models for aging research: (**A**) Primary Cells offer high physiological fidelity, but limited experimental control. They are derived directly from donors and possess inherent heterogeneity, biographical complexity (biological variation from different sources), and accumulation of epigenetic and metabolic memory. Manipulation of these cells is more constrained. Defining specific states of cells is limited to the selective elimination of specific subpopulations, such as senescent cells. “Steering” approaches using tools like Yamanaka factors or senolytics achieve partial and limited rejuvenation. This model is particularly valuable for studying authentic age-related pathology where cellular damage accumulation is essential and validating therapies in naturally aged cells. (**B**) Induced pluripotent stem cells (iPSCs) provide total experimental control, but at the cost of authenticity. The reprogramming process involves resetting cells to the embryonic state, a complete molecular reset that erases all hallmarks of aging. However, subsequent differentiation yields functionally immature cells (e.g., fetal neurons) that do not fully capture the adult pathophysiological function. To study aging, these cells can be subjected to artificial stressors to create an “artificially aged” phenotype. Although this does not fully recapitulate the multifaced aspect of cellular aging, it is valuable to understand the molecular signaling mechanisms.

In the following text, we discuss two different cellular systems used to investigate these phenomena—primary cells and induced pluripotent stem cells (iPSCs)—as summarized in Figure 2.

### 2.1. Primary Cells

Primary cell cultures offer a more physiologically relevant representation of cells in their natural environment compared to cell lines. However, primary cells have a finite lifespan and show significant variation among donors. This source variability can be attributed to differences in donor characteristics (age, sex, and health status), genetic and epigenetic factors, and prior environmental exposures. Nevertheless, primary cell cultures have been essential research tools for understanding human disease mechanisms, drug development, and toxicology.

One example is shown in striatal neurons derived from Huntington’s disease patient fibroblasts, which exhibit age-associated disease phenotypes such as DNA damage and neuronal loss [13], and neurons from aged donors that retain critical features of aging, including reduced mitochondrial activity and increased ROS levels [14]. However, it is notable that direct conversion efficiency inversely correlates with donor age, and this relationship varies by target cell type, showing reduced conversion efficiency from fibroblast to neuron in aged donors (~10–15% from aged vs. ~25–30% from young donors) [15], along with difficulty in directly reprogramming aged fibroblasts to cardiomyocytes [16].

Primary cells have played a crucial role in the initial identification of the senescence-associated secretory phenotype (SASP). Studies demonstrated that senescent cells communicate locally with their environment through a complex secretome enriched with inflammatory cytokines (such as IL-6 and IL-8), growth factors, and proteases (MMPs) that can contribute to tissue remodeling and age-related inflammation, respectively [17,18]. Additionally, these cultures have allowed for a comprehensive study of DNA damage response (DDR) mechanisms, including kinases like ATM (ataxia telangiectasia mutated) and ATR (ATM and Rad3-related), and downstream effector proteins such as p53 [19]. These molecules are critical in initiating and inducing the senescent state in response to various genotoxic insults [20,21]. Related to senescence, CAR T-cell therapy has emerged as a promising strategy for targeting and eliminating senescent cells, thereby potentially improving health span and treating age-related diseases [22]. CAR T-cells are engineered to express chimeric antigen receptors (CARs) that bind to specific surface markers of senescence, activating the T cells and directing them to eliminate the senescent cells.

The biological clock of primary cells is directly related to the donor’s age, epigenetic signature, and the passage number in culture. However, this information is often unavailable unless specifically tested for epigenetic markers. This is a limitation when using these cells to model aging or age-related diseases. In addition, recent single-cell RNA sequencing of primary fibroblast cultures has revealed that what was traditionally considered to be a homogeneous population actually contains distinct subpopulations with varying age. The aged spectrum includes the heterogeneity in proliferative, pre-senescent, metabolically stressed, pro-fibrotic, and quiescent cells [23]. This heterogeneity poses a challenge to the current paradigm of cellular aging, as it shows that a uniform cellular age cannot be defined within a population that contains multiple biological ages. Furthermore, recent studies using ATAC-seq and ChIP-seq have revealed tissue-specific aging signatures [24,25,26], metabolic scarring/memory in diabetic cells [27,28], inflammatory priming and trained immunity affecting SASP intensity [29], and inflammatory memory [30].

While we cannot select a specific state of aging for primary cells, sophisticated steering approaches are emerging, such as metabolic reprogramming, partial rejuvenation, epigenetic steering without identity loss, and proteostasis enhancement. For example, metabolic reprogramming with dichloroacetate aims to shift cellular energy production toward oxidative phosphorylation, showing promise in partially reversing age-related metabolic changes [31]. Similarly, targeted epigenetic modifications utilizing CRISPR-dCas9-TET1 systems have also demonstrated localized rejuvenation effects at aging-associated genomic loci [32,33].

In summary, although these in vitro approaches with primary cells offer directed therapeutic strategies, they have some limitations. Aging cells exhibit reduced metabolic flexibility, losing their capacity to efficiently switch between glycolytic and oxidative phosphorylation pathways [31,34]. While mitochondrial replacement from younger cells can restore certain cellular functions [35], it does not address aging mechanisms encoded in the nucleus [36]. These constraints suggest that comprehensive rejuvenation requires addressing multiple hallmarks simultaneously, which is currently very challenging.

### 2.2. Induced Pluripotent Stem Cells (iPSCs)

Induced pluripotent stem cell (iPSC) technology has been revolutionary for many human disease modeling studies [37,38]. iPSCs can be derived from any age or disease-state individual by reprogramming somatic cells with a defined combination of transcription factors, including OCT4, SOX2, KLF4, and MYC (OSKM, also known as Yamanaka factors). This reprogramming successfully rejuvenates the cells, erasing the markers of the epigenetic clock and effectively defining the embryonic state, regardless of the age of the cell source.

Yamanaka factors for rejuvenation and reprogramming present a fundamental paradox for aging research. While the reprogramming achieves a remarkable erasure of aging signatures—including complete reset of DNA methylation (e.g., epigenetic Horvath clock: 0 ± 2 years) [39], restoration of telomere length to an embryonic state (12–15 kb) [40], and restoration of youthful mitochondrial function [41]—this process also eliminates age-related features in the modeling of various late-onset diseases.

Reprogramming by Yamanaka factors effectively eliminates aging signatures, including epigenetic marks, chromatin modifications, and nuclear organization defects, proving that aging is not absolutely irreversible. Despite this molecular reset, iPSC-derived cells exhibit developmental immaturity that limits their applicability for aging research. For example, iPSC cardiomyocytes display embryonic-like action potentials and an underdeveloped contractile apparatus [42], while iPSC neurons show reduced morphological complexity and immature connectivity [43,44]. Aging may involve both erasable molecular damage and fixed developmental constraints, a conceptual framework that primary cell models cannot adequately explore, since they possess mature functionality, but accumulate progressive aging phenotypes.

Age-related diseases develop late in life. Because iPSCs resemble fetal cells after reprogramming, applying iPSCs to model late-onset diseases is challenging due to their immaturity. Given this limitation of iPSCs, a few studies have been conducted to artificially accelerate the aging process in iPSC-derived neurons by shortening their telomeres. Since telomere shortening is an essential hallmark of aging, this strategy was able to overcome this limitation partially. For example, human iPSCs derived from two patients with Parkinson’s disease were treated with a pharmacological inhibitor of telomerase, BIBR1532 [45], or overexpression of progerin (the truncated lamin A protein that causes Hutchinson–Gilford progeria syndrome) [46]. It successfully created human iPSC-derived dopamine neurons with significantly shorter telomeres. These artificially aged neurons exhibited several degenerative and senescent phenotypes, including the accumulation of mitochondrial reactive oxygen species (ROS) and DNA damage, in parallel with a progressive loss of tyrosine hydroxylase, the most critical enzyme in dopamine synthesis, which is impaired in Parkinson’s disease. These experiments provided proof of concept that modulating telomere length can induce age-related phenotypes in iPSC-derived neurons, yet these methods address only one hallmark of aging. In other words, they can only achieve partial aging, but not fully recapitulate the complex natural aging process.

Future directions emphasize refining these maturation and aging protocols and integrating iPSCs into more complex, physiologically relevant systems. The amenability of iPSCs to precise genetic editing, primarily using CRISPR-Cas9 technology, allows for the creation of isogenic cell lines to study the direct impact of specific genes on aging or disease phenotypes [47,48]. Genes implicated in longevity or age-related diseases can be manipulated to assess their functional consequences by differentiating iPSCs into human cell types of interest [49,50]. Given that iPSCs harbor the complete genetic information of the donor and iPSC-derived cells have the potential for autologous transplantation, thereby minimizing immune rejection, they are anticipated to play an increasingly significant role not only as models in aging research but also in future regenerative therapies targeting age-related tissue degeneration.

### 2.3. Stressors to Induce Aging in Cellular Models

No consensus or definitive evidence suggests a primary driver of in vivo cellular senescence and aging. Most likely, a combination of stressors, both intrinsic (e.g., replicative exhaustion and metabolic imbalance) and extrinsic (e.g., UV exposure and smoking), that occur with aging, or a related chronic disease, contribute to the cumulative senescence burden of the organism [51]. In a cell culture model, cellular senescence can be driven by a diverse set of senescence-inducing stimuli, including genotoxic stresses (ionizing radiation, doxorubicin, etc.), oncogenic stress (RAS activation, etc.), metabolic stress (mitochondrial dysfunction), oxidative stress (ROS), and others [51,52,53]. Interestingly, certain treatments that patients take for conditions such as cancer (e.g., doxorubicin chemotherapy) [54] or HIV (e.g., the protease inhibitor atazanavir) [18] can drive cells into senescence in culture, suggesting that cellular senescence is clinically relevant in the context of patients undergoing these therapies. This idea has been supported by preclinical models, suggesting side effects of chemotherapy could be mitigated via clearance of senescent cells [55].

When designing a cell culture model of cellular senescence, there are several key steps, including (1) model selection, (2) control condition selection, (3) induction of senescence and control conditions, and (4) validation of senescence induction. For a detailed protocol covering these steps, refer to Neri et al. [56].

Genotoxic stress models represent the most standardized approach, with ionizing radiation at 10–15 Gy remaining the gold standard for generating uniform senescent populations in primary fibroblasts, requiring 7–10 days for phenotype development [54,57,58]. Chemical alternatives such as etoposide and doxorubicin offer dose-controlled induction with tissue-specific relevance, particularly useful when modeling specific clinical scenarios like doxorubicin-induced cardiotoxicity in cardiac cells. Stress-induced premature senescence (SIPS) protocols utilize acute stressors, including H_2_O_2_, DNA-damaging agents, or oncogene overexpression, to trigger rapid senescence via p53/p21 or p16/Rb pathways. SIPS approaches are preferred when modeling acute stress responses or when standard protocols are incompatible with the cell type under study [59]. Specialized models include progeroid syndrome cells such as HGPS fibroblasts, which model specific defects in aging mechanisms, such as genomic stability and nuclear architecture, rather than general senescence mechanisms. These systems are particularly valuable for studying particular aging pathways rather than broad senescence responses [60].

If the goal of a study is to identify senescence-associated features (proteins, transcripts, etc.), the control condition will have a substantial impact on the results. If comparing senescent and proliferating cells, many of the differentially expressed proteins will be cell cycle-related. However, these proteins may not be ideal for distinguishing quiescent cells from senescent cells. More robust and specific markers of senescence are obtained by including multiple or carefully selected controls. Importantly, control conditions are not uniformly applied in the literature and may drive differences in reported “senescence-associated” phenotypes. Like senescence models, induction of comparative control conditions should be tailored to the model under examination. For example, in some cell types, contact inhibition may be a preferred model for quiescence induction, as opposed to serum starvation, to maintain consistent serum supplementation between senescent and control conditions.

Since no single marker defines all senescent cells, validation requires markers spanning multiple hallmarks of senescence. As per the latest guidelines from the Cellular Senescence Network (SenNet) consortium [61], a minimum adequate panel should include a proliferation assay such as bromodeoxyuridine (BrdU) incorporation to demonstrate growth arrest, canonical markers p16 or p21 for cell cycle assessment, β-galactosidase activity for lysosomal changes, and SASP components such as IL-6 or GDF15. Context-specific additions may include γH2AX for DNA damage assessment and morphological evaluation for cellular changes. The validation panel should be expanded when developing novel protocols or studying heterogeneous cell populations where marker expression may be variable.

Interventional approaches provide functional validation of senescence models. Senolytic compounds like dasatinib plus quercetin (D+Q) or Fisetin, which selectively eliminate senescent cells often by targeting pro-survival pathways (e.g., BCL-2 family proteins) [62,63]. Telomerase activation through TERT overexpression can extend replicative lifespan in telomere-limited cells [64,65]. Cellular aging models require careful consideration of stressor selection, appropriate controls, and comprehensive validation. The choice of experimental system should be guided by the objectives of the research and the biological relevance of the phenomenon under investigation.

### 2.4. Epigenetic Alterations in Cellular Models

The epigenetic modifications that emerge from induced aging protocols represent a critical intersection in cellular aging research. Unlike transient stress responses, these chromatin changes establish stable molecular memories that persist long after the initial insult, fundamentally constraining future cellular behavior and intervention possibilities.

The enzymatic apparatus that controls these changes constitutes the mechanism of steering as well as the targeting approaches, because various stressors mediate different pathways of regulation. Genotoxic treatments mainly target DNA methyltransferases (DNMTs) and ten-eleven translocation (TET) enzymes by activating DNA damage response and consequently generating the methylation imbalance that defines senescent cells. Oxidative stress directly disrupts the catalytic function of TET enzymes by oxidizing their iron cofactors and simultaneously activates stress-responsive histone acetyltransferases such as KAT7 [66]. Oncogenic stress engages different pathways, driving SIRT1 and SIRT6 deacetylase through inflammatory signaling while activating specific histone methyltransferases [67]. Metabolic stress creates substrate limitation for chromatin-modifying enzymes, reducing SAM availability for methylation reactions while depleting NAD+ levels required for sirtuin activity [68,69]. These stress-specific enzymatic disruptions suggest potential intervention points, but current approaches remain limited to broad enzymatic modulation rather than precise targeting of age-related modifications.

The development of predictable DNA methylation-based epigenetic clocks using cells exposed to different stressors reveals both the conservation and divergence of aging signatures. While certain clock CpG sites respond similarly to genotoxic treatments, oxidative stress, and metabolic stress, stress-specific methylation patterns exist that may require distinct reversal strategies. This suggests that successful epigenetic defining will need to account for the historical stress exposure profile of cells rather than assuming universal aging signatures.

The potential clinical implications are whether therapeutic epigenetic defining can be accomplished, given the complexity and stress specificity of these regulatory networks. Although reversal of individual epigenetic marks is conceptually possible by specific enzymatic activity, the practicality is the development of interventions capable of negotiating the brewing chromatin, RNA, and enzyme networks that define the distinct aging stressor patterns. Understanding these comprehensive, stress-specific epigenetic consequences thus frames both the promise and limitations of precision aging interventions, highlighting the need for personalized approaches that account for individual stress exposure histories before transitioning from broad cellular steering to targeted epigenetic defining becomes experimentally and clinically feasible. A summary of representative molecular interventions for modulating cellular aging in vitro, including their targets, mechanisms, and model applications, is provided in Table 1.

### 2.5. Progeria or Premature Aging Cells

Patients with progeroid syndromes display several features of premature or accelerated aging involving specific tissues. There are at least two categories based on the underlying molecular pathogenesis: first, mutations of proteins involved in nuclear envelope stability and organization, such as nuclear lamins, as in Hutchinson–Gilford progeria syndrome (HGPS); and second, mutations in DNA damage repair pathways, such as Werner syndrome, xeroderma pigmentosum, Cockayne syndrome, Bloom syndrome, ataxia telangiectasia, and Fanconi anemia, etc. Given the difference in the genes or pathways involved in these progeria syndromes, there are variations in the clinical spectrum and the extent of the affected tissues. Nevertheless, cells from the affected tissues of these progeria syndromes demonstrate several hallmarks of aging, including genomic instability, epigenetic alterations, telomere attrition, loss of proteostasis, and impaired autophagy. Accumulation of DNA damage and the activation of downstream checkpoint kinases are common findings in both chronological aging and accelerated damage in progeroid syndromes. These mainly contribute to the development of cellular senescence, which is a permanent arrest in cell cycle. Therefore, when using the cells derived from patients with progeria syndrome for aging research, it should be kept in mind that these senescent cells have slow and very limited proliferative capacity. Many will stop dividing (a complete cell cycle arrest state) at a very low passage number, thus limiting the supply for aging research. However, given that these are clinically relevant patient-derived cells, they are invaluable for testing particular pathways that may restore or reactivate the cell cycles.

Other hallmarks of aging not directly caused by genetic mutations of progeria syndrome are also present. For example, SASP-related chronic inflammation was reported in adipose tissue from Werner syndrome patients [89]; altered intercellular communication induced myofibroblast switch-promoting fibrosis in cardiovascular tissues of HGPS [90]; and stem cell exhaustion leading to impaired tissue regeneration and repair capacity was observed in HGPS [91], Werner syndrome [92], and Cockayne syndrome [93]

Moving beyond human cellular models, mouse models with progeroid phenotypes, such as a mutation in mitochondrial polymerase gamma (Polg) [94,95] and homozygous hypomorph of α-klotho [96], provide additional context for understanding progeria mechanisms. Interestingly, mice with homozygous mutation of the proof-reading capacity of mitochondrial polymerase gamma (PolgaD257A) demonstrate features of “accelerated aging phenotypes” [94], including alopecia, graying, kyphosis, osteopenia, sarcopenia, and age-dependent cardiomyopathy [95]. Importantly, the phenotypes of Polg-mutant mice differ from the clinical spectrum of human POLG patients, who primarily develop encephalopathy and muscle weakness [97]. Instead, this clinical spectrum of POLG patients highly overlaps with other mitochondrial diseases, different from progeria syndrome. The second model, mice with homozygous hypomorph of α-klotho showed features of “accelerated aging” including skin and muscle atrophy, osteoporosis, and premature death at around 8–12 weeks of age [96], as well as hyperphosphatemia and dystrophic vascular calcification, which resembles chronic kidney diseases with derangement of calcium and phosphate metabolism [98]. Klotho as an “antiaging hormone” is supported by the fact that the circulating levels of α-klotho declines with age in mice [99] and humans [100]. In contrast, supplementation of α-klotho is beneficial to rejuvenate cardiac aging and diastolic dysfunction [99] as well as reactivate the cell cycle re-entry in progeroid cells. The downstream mechanisms of α-klotho are complex, including the activation of Sirt1, NRF2 [100], and Ca signaling.

## 3. In Vitro Approach to Model Organ Aging

### 3.1. Cardiac Aging

Understanding the molecular mechanisms and signaling pathways involved in cardiovascular aging is essential for developing interventions to slow the aging process in the cardiovascular system and to treat age-related cardiovascular diseases. Crucial aging mechanisms include mitochondrial oxidative stress and dysfunction, nutrient signaling (mTOR), altered proteostasis, epigenetic shifts, senescence, etc. All of these mechanisms also play critical roles in the development of heart failure and various other heart diseases. Mammalian hearts comprise a diverse range of cell types, including cardiomyocytes, fibroblasts, endothelial cells, vascular smooth muscle cells, immune cells, and valvular interstitial cells. Most of these cell types are implicated in cardiac aging processes, although a great majority of previous studies have focused on cardiomyocytes.

Most prior research has investigated cardiac aging in vivo. Rodents, in particular mice, have been extensively used in cardiac aging research [101,102,103]. The Drosophila model has also been valuable in elucidating signaling mechanisms in cardiac aging [104,105]. Due to their physiological and anatomical resemblance to humans, many large mammals have been utilized to investigate specific age-related human cardiovascular diseases. Pigs’ and dogs’ hearts have similar electrophysiological properties to human hearts, making them suitable models for studying various cardiac arrhythmias. Non-human primates (NHPs) are particularly valuable for studying coronary heart disease and regenerative medicine due to their closer anatomical resemblance to humans.

This section aims to explore modeling age-related cardiac phenotypes using cardiac cells. In vitro “cardiac aging” models may apply either primary cells or iPSC-derived cardiovascular lineages. We have discussed stress-induced senescence above. Exposing cells to various stressors, such as mitochondrial toxins and oxidative stress (e.g., doxorubicin treatment), or genotoxic stress can induce senescence resembling age-related damage. Maintaining cells in culture for extended periods can induce an aging phenotype, a replicative senescence characterized by decreased cell proliferation, accumulation of DNA damage and mitochondrial oxidative damage, higher expression of senescence markers (e.g., p16, p21, and SA-β-galactosidase), and DNA methylation changes. The CRISPR-Cas9 genetic editing tool has been used to induce telomere shortening. Since telomere length shortens with age, this strategy can induce senescence and model age-related cellular changes. Culturing cells in aged extracellular matrix or media enriched in SASP increases exposure to factors within an aged microenvironment, which can lead to age-associated changes.

A few examples of studies using in vitro methods are discussed in the following. Mitochondrial therapies have been used to reverse age-related decline in mitochondrial function within cardiac cells based on several of our mouse studies [101,106]. These approaches include delivering NAD+ precursors, such as nicotinamide mononucleotide and nicotinamide riboside, to boost intracellular NAD+ levels. Elevated NAD+ can enhance the activity of NAD+-dependent enzymes, such as sirtuins (SIRT1) [99,107], which play roles in deacetylating mitochondrial proteins, improving mitochondrial quality control via mitophagy, and supporting overall metabolic health [108,109]. Other strategies focus on agents that promote mitochondrial biogenesis, such as activators of PGC-1α (peroxisome proliferator-activated receptor gamma coactivator 1 alpha), like resveratrol, in certain experimental contexts [110,111].

Pharmacological intervention is another major approach using specific small-molecule inhibitors or activators that directly act on the vital signaling nodes involved in cardiac aging. The most prominent examples are mTOR inhibitors (e.g., rapamycin and everolimus) [112], which appear to be able to reproduce some of the benefits of caloric restriction while also altering the conduct of autophagy and possibly preventing pathological hypertrophy [103,113]. Other examples include AMPK activators, such as metformin and AICAR, which increase mitochondrial capacity and improve resistance to stress by making the cell more sensitive to its energy status [114]. There are also targeted HDAC inhibitors that could reverse detrimental epigenetic programs associated with cardiac hypertrophy or fibrosis [115]. Controlled in vitro conditions are especially beneficial for such studies, permitting the definition of direct cellular effects through the detailed analysis of dose–response relationships and the temporary regulation of pathway modulation. In addition, strategies to inhibit the hallmarks of aging using small molecules have been developed. Examples include compounds that selectively improve proteostasis, such as chemical chaperones that inhibit 4-phenylbutyrate 72 (4-PBA), which can attenuate ER stress, activators of individual branches of the unfolded protein response (UPR), or integrated stress response inhibitor (ISRIB) [116,117]. Likewise, compounds that augment chaperone-mediated autophagy (CMA) or bulk macroautophagy, such as spermidine (which also modulates eIF5A hypusination* and EP300 activity) [118], are administered to cultured cardiac cells, and the efficacy of such enhancing activities in reducing protein aggregations, improving mitochondrial quality, and maintaining cardiac cell survival and functional/physiological effectiveness, particularly under stress, is quantified. The application of senolytic compounds, such as dasatinib and quercetin (D+Q) or the flavonoid fisetin, to cardiac cultures represents a strategy of selective elimination of damaged cells. When D+Q is applied to aged murine cardiac fibroblasts, senescent cell clearance occurs within 48–72 h, reducing SASP burden by 60–70% [70]. This represents removal of dysfunctional cells to avoid further damage, but not a direct rejuvenation of the remaining population [119,120].

It is noteworthy that primary cardiomyocytes have a short lifespan (hours for isolated adult CM and days for isolated neonatal CM in regular culture media), thereby limiting their utilization to model aging. In this context, iPSC-derived CM provide an invaluable tool to study signaling pathways in aging and senescence. Other cardiac cell types may use either primary cells (e.g., endothelial cells, smooth muscle cells, and cardiac fibroblasts) or iPSC-derived cells. The latter may have disadvantages of higher cost and a less mature state. In addition to individual cell types, engineering cardiac tissue using iPSC-derived 3D cardiac tissues supported by scaffolds, or cardiac organoids, may enable the study of cardiac aging in a more physiologically relevant context, representing an innovative method for in vitro modeling using human cells.

CRISPR-Cas9 technology allows for precise editing of the genome in iPSC-derived cardiac cells, including iPSC-CMs and iPSC-CFs [121,122]. Loss-of-function studies may involve knocking out genes known to promote aging or specific age-related cardiac pathologies. These studies can target certain genes essential for the development of fibrosis (e.g., TGFBR1 (receptor for TGFB), or CTGF (connective tissue growth factor)), major pro-inflammatory signaling molecules (e.g., the components of the NLRP3 inflammasome) [123,124,125], or specific epigenetic modifiers (e.g., certain histone methyl- or demethylases) whose activity is linked to premature aging [126]. The goal is to assess if ablation of these factors prevents or slows the acquisition of age-related phenotypes during intrinsic aging through serial passaging or in response to extrinsic stressors in vitro. Conversely, gain-of-function experiments involve overexpressing putative protective genes. These might include specific sirtuins like SIRT1 or SIRT3 [127], known to enhance mitochondrial function and stress resistance, antioxidant enzymes such as mitochondrial superoxide dismutase (SOD2) [128] or catalase [101], or TERT in contexts where telomere shortening is a limiting factor, for instance, in certain cardiac progenitor populations [78,129]. The goal is to evaluate whether these regulators can protect cardiac cells from age-associated stress, slow senescence, or enhance specific cellular functions, such as contractility or metabolic flexibility.

Future studies may combine iPSC-derived cardiac cells or 3D cardiac tissues combined with CRISPR-Cas9 genetic modifications of interest using well-known stressors to induce senescence and aging-related pathways and test the efficacy of small-molecule inhibitors to attenuate the phenotypes and signaling pathways in cardiac aging.

### 3.2. Brain Aging

The brain primarily consists of postmitotic cells with precise functional capabilities. Disease models, particularly those related to neurodegenerative diseases and mitochondrial disorders, serve as critical tools for understanding cellular phenotypes, signaling mechanisms of brain aging, and testing the feasibility of various interventions.

Declining proteostasis is a common feature of aging and many neurodegenerative diseases. In vitro systems have been extensively applied to examine toxic protein aggregates and the proteostasis pathway. iPSC-derived neurons from patients with Alzheimer’s disease (AD) or Parkinson’s disease (PD) or directly engineered cell lines ex-pressing amyloid beta (Aβ), hyperphosphorylated tau, or α-synuclein can be used to test compounds or biological agents that stimulate the reversal of aggregation or improve clearance. These interventions may include methods that enhance macroautophagy or lysosomal activity through mTOR inhibitors like rapamycin [130] and compounds such as trehalose that stabilize proteins and induce autophagy [131,132] or directly modulate protein aggregates. However, many of these interventions result in partial restoration, but never complete reversal, indicating that postmitotic neurons have intrinsic limitations regarding proteostasis rejuvenation.

Partial epigenetic reprogramming has emerged as a compelling strategy for rejuvenation. This approach involves the transient expression of Yamanaka factors (OSKM) in aged or diseased iPSC-derived neurons and glia in vitro [133,134], with the potential to reset epigenetic clocks and restore more youthful gene expression profiles related to synaptic plasticity or neurotrophic factor production. It also aims to improve mitochondrial function and enhance resilience to neurotoxic insults, all without complete dedifferentiation and loss of cellular identity. In parallel, the rejuvenation of neural stem cells (NSCs) focuses on addressing their age-related exhaustion, which is a well-recognized hallmark of aging [135,136].

These pursuits raise important questions, particularly in the context of neural networks. For neurons, defining “rejuvenation” beyond functional restoration is challenging. Does a molecular reset, such as partial epigenetic reprogramming, truly equate to a younger neuron if its intricate synaptic connections, shaped by lifelong experience, remain largely intact or are subject to the intervention itself? Additionally, the stability of any rejuvenated state in vitro, and more critically whether it can be translated into meaningful and sustained cognitive or motor improvements in vivo, during organismal aging remains unsolved. In the case of NSC-based strategies, while the prospect of generating “new” neurons is enticing, significant challenges remain in ensuring their proper integration into existing complex network of neural circuits, achieving functional maturation, and ensuring long-term survival in the aged-brain environment.

Many signaling mechanisms are conserved in aging and diseases, particularly those in mitochondrial disease models. The shared mechanisms include nutrient and metabolism, mitochondrial respiration, dynamics, and autophagy. Indeed, models of dysfunctional mitochondria provide invaluable insights into how energetic failure drives nervous system aging. For example, Leigh syndrome is a severe mitochondrial disease characterized by early-onset neurodegeneration, various cardiomyopathies, and motor dysfunction resulting from mutations involving mitochondrial respiratory chain complexes, particularly complexes I, IV, and V. This results from mutations in mitochondrial or nuclear genes that encode components of the mitochondrial respiratory chain or electron transport chain (ETC). The ETC defects impair electron transfer, leading to energy deficiency and disrupted mitochondrial signaling. Although the initiating causes differ (genetic mutation vs. accumulated damage), both conditions culminate in very similar downstream outcomes. For instance, the decline in mitochondrial complex I and the increased ROS production in aging resemble those observed in mitochondrial complex I deficiency observed in Leigh syndrome. Furthermore, the resultant proteotoxic stress responses activated in both aging and mitochondrial diseases, such as mitochondrial unfolded protein response (UPRmt) and integrated stress response (ISR), are similar. The rapid progression of neurodegeneration in Leigh syndrome may be useful as a short-term model for the aging process that occurs over decades. The clonal expansion of mitochondrial mutations also aligns with age-related selective neurodegeneration [137], demonstrating compensatory mechanisms and vulnerability patterns across different cell types [138]. This approach mirrors the use of progeroid syndromes to gain insights into aging, not because they perfectly replicate the aging process, but because they illuminate fundamental mechanisms at a much faster pace (not decades of time required for normal aging). One insight from studying Leigh syndrome is that aging does not equate to mitochondrial disease: rather, understanding cellular responses to severe bioenergetic stress in mitochondrial disease reveals potential pathways to enhance resilience against age-related mitochondrial decline.

The advancement of iPSC technology has enabled the modeling of mitochondrial diseases, including Leigh syndrome. These iPSCs can recapitulate several hallmark phenotypes observed in aging research. Therapeutic agents developed for antiaging intervention, such as NAD+ supplementation, sirtuin activators, mitochondrial antioxidants and rapamycin, have been tested in Leigh syndrome models, including Ndufs4-deficient mice and iPSC-derived cardiomyocytes and neurons, with promising outcomes in restoring ATP production, reducing oxidative stress, and improving cell survival [76,139,140,141,142]. By “steering” damaged cells toward improved survival states, interventions like mTOR inhibition or enhancing mitochondrial proteostasis can mitigate damage accumulation and extend tissue function in the context of pathogenic mutations in mitochondrial components [76,143]. As such, Leigh syndrome not only deepens our understanding of mitochondrial pathophysiology but also provides a platform to refine rejuvenation strategies targeting mitochondrial aging for both rare diseases and broader age-related conditions. In addition, iPSCs can be differentiated into various resident cells in the brain for precise characterization of neuronal, glial, and vascular cells to dissect the causal contributions of specific genes and pathways within specific cell types. One objective is to guide these cells towards states that are more resilient or functional in a healthy context or resistant to disease-specific insults.

Furthermore, gene- and base-editing tools like CRISPR-Cas9 and TALEN enable the generation of isogenic controls, allowing researchers to dissect the direct consequence of specific mitochondrial gene mutations on cellular aging and disease phenotypes [144,145,146]. iPSCs can differentiate into multiple types of cells and organoids, serving as a powerful tool of modeling aged cells (e.g., cardiomyocytes and neurons) or tissues (e.g., cardiac organoids, engineered heart tissues, brain organoids, and others). However, when creating an aging model from mitochondrial mutants, it is essential to acknowledge that heterogeneity is a prominent feature, and not all cell types will demonstrate distinct phenotypes.

One example applying CRISPR-Cas9 technology is to generate loss-of-function experiments by inducing pathogenic mutations or disease-risk alleles. For instance, editing the APOEε4 allele to the more common APOEε3 variant in iPSC-derived neurons and astrocytes can provide insights in Aβ metabolism and neuroinflammation [147], while correcting mutations in LRRK2 or SNCA can be applied to PD models [148]. Conversely, gain-of-function approaches involve overexpressing potentially neuroprotective factors such as longevity-associated sirtuins (e.g., SIRT1, which has roles in DNA repair, mitochondrial function, and proteostasis), components of the antioxidant response like transcription factor NRF2 or its target genes like HMOX1, and specific heat-shock proteins that act as molecular chaperones to combat proteotoxicity, enhancing neuronal survival and synaptic health in culture [149,150]. Moreover, a disease-specific state can also be engineered by modifying α-synuclein to include a light-responsive domain (Cry2clust), enhancing rapid and controllable aggregation of α-synuclein into forms that mimic the pathological aggregates found in PD [151].

## 4. Conclusions

Aging is a multifaceted and complex process. In this review, we discussed the application of in vitro cellular models to understand the molecular mechanisms of aging. This review explored how these in vitro systems are used for modeling the complex cross talk between chronological and biological time, and serves as a critical proving ground for tactics developed to “steer” the aging of cells. We covered how stressors can cause accelerated senescence and how both genetic and pharmaceutical approaches can be used to modulate specific pathways in stress resistance. We also discussed how epigenetic reprogramming promises the enticing possibility of cellular rejuvenation. With a focus on cardiovascular and neurodegenerative diseases, we reviewed how these cell platforms allow for the study of age-associated pathologies in disease-relevant settings, shifting from an observer-based approach to an actor-based approach. The ability to create models for progeroid syndromes and mitochondrial diseases, such as Leigh syndrome, further emphasizes the strength of these models for investigators desiring to study accelerated aging and to test interventions with a broader range of applications.

Current technologies, even the most aggressive rejuvenation protocols, often achieve only a partial and transient reversal of the aged state. The rapid reversion of cellular phenotypes upon cessation of treatment suggests that many interventions temporarily suppress aging programs rather than erase them completely. This is why the choice of cellular model is critical. For studying durable functional rescue, models that retain cellular maturity and an aged phenotype, such as primary cells or directly reprogrammed cells, are indispensable. However, their limitations are often a finite supply and inherent biological variability.

## 5. Perspective

This review highlights the paradox and the specific utility of iPSCs in aging research. If iPSCs are developmentally immature or “arrested,” how can they be useful for modeling an age-related process? Their value lies precisely in their capacity for an almost complete epigenetic marker reset. The reprogramming process itself erases epigenetic and other molecular hallmarks of aging, providing a “clean slate” from which to study the mechanisms of aging. While their immaturity makes them challenging models for testing the restoration of complex, mature-cell functions, iPSCs are unparalleled platforms for dissecting the molecular pathways that govern the reacquisition of age-related biomarkers and for testing interventions that aim to prevent this decline de novo.

Current transformative strategies, such as senolytic therapies like CAR-T cells that target senescent cells, could achieve a form of cell elimination rather than cell transformation. They selectively clean up damaged cells, avoiding the contagious effects of SASP from causing harm to the neighboring cells, providing a healthier microenvironment. However, the surviving cells do not rejuvenate to a new, younger age. Future challenges may include bridging the gap between resetting molecular biomarkers and achieving durable functional rejuvenation in an organism, i.e., a successful and controllable reprogramming to achieve a youthful state without affecting cell identity (differentiation status). A multimodality approach leverages the unique strengths of each model system: iPSCs for mechanistic discovery on a rejuvenated background, primary and directly converted cells for testing functional rescue in an aged context, and advanced 3D organoids and organ-on-a-chip systems to better recapitulate physiological complexity.

## Figures and Tables

**Table 1:** Selected interventions for modulating cellular aging.

Intervention Strategy	Compound/Factor	Primary Target/Mechanism	Model System Application	Validation Status	References
Senolysis	Dasatinib + Quercetin (D+Q),	Induce apoptosis in senescent cells by inhibiting BCL-2 pathways	Aged primary cells, iPSC-derived cultures, aged animal cells	Clinically validated Ongoing phase II (NCT04685590)	[70]
	Fisetin		Aged primary cells, progeria models	Conceptual	[71]
Epigenetic Reprogramming	OSKM (Oct4, Sox2, Klf4, Myc)	Reset epigenetic age, restore gene expression, elongate telomeres	Aged fibroblasts, iPSC-derived cardiomyocytes	Conceptual	[72]
	Transient OSKM Expression	Partial reprogramming reverses aging markers without identity loss	Aged fibroblasts, human cell lines	Conceptual	[73]
mTOR Inhibition	Rapamycin, Everolimus	Inhibits mTOR, modulates autophagy	Primary cardiac cells, iPSC-derived neurons	Clinically validated Phase I completed (NCT04488601)	[74,75,76]
AMPK Activation	Metformin, AICAR	Activates AMPK, enhances mitochondrial function	Primary cardiac cells	Clinically validated Ongoing phase II/III (NCT06459310)	[77]
Telomere Maintenance	hTERT	Counteracts telomere shortening	Fibroblasts, cardiac progenitor cells	Conceptual	[78]
Sirtuin Activation	Resveratrol, NAD+ Precursors (NR, NMN)	Activates SIRT1, SIRT3, enhances mitochondrial health	iPSC-derived cells, cardiac models	Clinically validated Ongoing phase II/III (UMIN000030609)	[79]
	MDL-800 (SIRT6 Activator)	Activates SIRT6, enhances DNA repair and reduces senescence	Human fibroblasts, murine-derived iPSCs	Conceptual	[80]
Proteostasis Enhancement	Spermidine, 4-PBA, ISRIB	Improves protein quality control, reduces ER stress	Cardiac cells, neurons	Conceptual	[81]
HDAC Inhibition	Various HDAC inhibitors	Reverses epigenetic programs, reduces fibrosis	Cardiac cell models	Conceptual	[82]
KAT7 Inhibition	Specific KAT7 inhibitors	Targets histone acetyltransferase implicated in senescence	Senescent cells in culture	Conceptual	[83]
Mitochondrial Protection	MitoQ, SkQ1, Elamipretide	Reduces mitochondrial ROS, improves mitochondrial function	Primary fibroblasts, neuronal cells, cardiomyocytes	Conceptual	[84,85,86,87]
Senomorphic	Metformin, Rapamycin	Modulates SASP, reduces inflammation without cell death	Aged primary cells, iPSC-derived models	Clinically validated Ongoing phase II (NCT03359538)	[75,88]
